# A distinct symptom pattern emerges for COVID-19 long-haul: a nationwide study

**DOI:** 10.1038/s41598-022-20214-7

**Published:** 2022-09-23

**Authors:** Melissa D. Pinto, Charles A. Downs, Yong Huang, Sarah A. El-Azab, Nathan S. Ramrakhiani, Anthony Barisano, Lu Yu, Kaitlyn Taylor, Alvaro Esperanca, Heather L. Abrahim, Thomas Hughes, Maria Giraldo Herrera, Amir M. Rahamani, Nikil Dutt, Rana Chakraborty, Christian Mendiola, Natalie Lambert

**Affiliations:** 1 Sue & Bill Gross School of Nursing, University of California Irvine, Irvine, CA USA; 2 University of Miami, Coral Gables, FL USA; 3 Department of Computer Science, Donald Bren School of Information and Computer Science, University of California Irvine, Irvine, CA USA; 4 Department of Health Management and Policy, University of Michigan, Lansing, MI USA; 5 Brown University, Providence, RI USA; 6 School of Labor and Employment Relations, University of Illinois at Urbana-Champaign, Champaign, IL USA; 7 Lambert Health Lab, Indiana University School of Medicine, Indianapolis, IN USA; 8 Electrical and Computer Engineering Department, Indiana University Purdue University Indianapolis, Indianapolis, IN USA; 9 Division of Infectious Disease, Department of Pediatrics and Adolescent Medicine, Mayo Clinic, Rochester, MN USA; 10 School of Engineering and Technology, Indiana University Purdue University Indianapolis, Indianapolis, IN USA; 11 Department of Biostatistics and Health Data Sciences, Indiana University School of Medicine, Indianapolis, USA

**Keywords:** Computational biology and bioinformatics, Risk factors, Signs and symptoms

## Abstract

Long-haul COVID-19, also called post-acute sequelae of SARS-CoV-2 (PASC), is a new illness caused by SARS-CoV-2 infection and characterized by the persistence of symptoms. The purpose of this cross-sectional study was to identify a distinct and significant temporal pattern of PASC symptoms (symptom type and onset) among a nationwide sample of PASC survivors (n = 5652). The sample was randomly sorted into two independent samples for exploratory (EFA) and confirmatory factor analyses (CFA). Five factors emerged from the EFA: (1) cold and flu-like symptoms, (2) change in smell and/or taste, (3) dyspnea and chest pain, (4) cognitive and visual problems, and (5) cardiac symptoms. The CFA had excellent model fit (*x*^2^ = 513.721, df = 207, *p* < 0.01, TLI = 0.952, CFI = 0.964, RMSEA = 0.024). These findings demonstrate a novel symptom pattern for PASC. These findings can enable nurses in the identification of at-risk patients and facilitate early, systematic symptom management strategies for PASC.

## Introduction

Long-haul COVID-19, post-acute sequelae of SARS-CoV-2 (PASC), is the persistence of symptoms that extend beyond the expected resolution of illness^1^. PASC often causes significant disability and given the large-scale of the pandemic coupled with the lack of treatments, PASC is a global health crisis^2,3^. PASC has not yet been clinically characterized. The incidence PASC is estimated 11 to 80%^4–9^. Initial COVID-19 symptom presentation varies; however, the development of PASC appears to be independent of COVID-19 symptom presentation, severity, or the presence of pre-morbid (pre-existing) health conditions^10^. Persons at risk for PASC include those who were initially asymptomatic, as well as symptomatic persons independent of needing hospitalization^5,11,12^. PASC survivors report a variety of persistent and distressing symptoms that can last anywhere from weeks to more than a year^10^. Nearly three years into the pandemic, PASC survivors continue to report symptoms and it is unclear if symptoms will eventually resolve or if a new chronic disease has emerged. We and others have numerated and characterized PASC symptoms at the onset of illness^5,10,11,13–15^. However, it is still not known how PASC symptoms evolve over time, how they temporally group or cluster, and if findings represent a statistically significant pattern.

PASC symptom presentation, and more specifically the order in which symptoms are experienced, is useful for developing and implementing self-management strategies to reduce symptom burden and improve quality of life and daily functioning. As experts in symptom science, nurse scientists are well-poised to investigate PASC symptoms and leverage the existing self-management evidence base to develop or re-tool interventions to mitigate the devastating consequences of PASC symptoms^10^. Further, understanding the PASC symptom pattern allows for the exploration of the underlying biological mechanisms underpinning symptoms and their evolution^10^. Understanding symptoms and their context (i.e., biology, behaviors, etc..) is critical for the development of new interventions and pharmacological and non-pharmacological therapeutics. This study extends beyond symptom description and advances our understanding of PASC symptoms through added context, specifically symptom onset. Therefore, the aim of this study was to identify a distinct and statistically significant pattern of PASC symptoms through assessment of symptom type and symptom onset in a nationwide sample of PASC survivors.

## Methods

### Sample and setting

A convenience sample (n = 5562) of COVID-19 survivors were recruited (August 2020 through February 2021) through online COVID-19 survivor groups and online COVID support communities in response to a written study advertisement. Since study recruitment and data collection were completed online, the sampling frame was national. Inclusion criteria were English speaking, adults at least 18 years of age or older, no history of hospitalization for SARS-CoV-2 infection, and either a (self-report) positive PCR or history of healthcare provider confirmed clinical diagnosis of SARS-CoV-2 infection. Exclusion criteria were non-English speaking, less than 18 years of age, and hospitalization for SARS-CoV-2 infection.

### Survey and procedures

Institutional review board (IRB) approval was obtained from Indiana University prior to recruitment and data collection. Informed consent as approved by the IRB was implied prior to the completion of the survey. All methods were performed in accordance to the Declaration of Helsinki. Since there is no gold-standard symptom survey measure for PASC, a symptom survey was developed using unstructured social media data in which patients reported their symptoms in free text. Further details regarding survey development are reported in^15^. The survey asked participants to indicate (check) symptoms they had experienced since the onset of COVID-19 and at the time of completing the survey. Participants were then asked to write in symptom onset (the number of days after SARS-CoV-2 infection that a symptom began) of symptoms they checked. A free text option was included in which participants could report any symptom not included in the survey. Symptoms reported in the free text were evaluated for consistency across participants and collapsed into common categories as appropriate. PASC is operationalized as experiencing symptoms more than 28 days after SARS-CoV-2 infection. All data collection were completed electronically using REDCap.

### Analytic plan

Exploratory factor analysis (EFA) and confirmatory factor analysis (CFA) were used. EFA was used to identify the underlying PASC symptom structure (symptom type and onset) and the temporal clustering of symptoms. CFA was used to validate findings of the EFA PASC symptom structure, and thereby yielded a distinct, statistically significant temporal pattern of symptoms among PASC survivors.

Statistical analyses were performed using R software (Version psych 2.1.6 (EFA) and Lavaan 0.6–9 (CFA)). To ensure group equivalence descriptive statistics and measures of central tendency and *t-test* were used to describe the sample, test for differences at baseline, and onset of symptoms.

A sample of 5,562 participants were recruited, and 5136 met criteria. The sample (n = 5136) was randomly sorted into two independent samples for EFA (n = 2547) and CFA (n = 2589) analyses using a random number generator. The sample size exceeded the minimum criteria for subjects per item (10:1) ratio needed to conduct rigorous exploratory and confirmatory factor analyses^16^. The following criteria were used to guide the exploratory and confirmatory factor analyses:

### Exploratory factor analysis

#### Extraction and rotation method

A series of EFAs were performed using R to determine the factor structure of symptom onset for PASC. Latent factor structure was assessed through principal axis factoring (PAF) to extract the symptom onset for PASC factor structure. PAF accounts for the unique contribution of each item and can robustly analyze the data that violate the assumption of normality. Prior to the EFA, indicators of sampling adequacy were verified by Kaiser–Meyer–Olkin (KMO) and Bartlett’s Test of Sphericity^16^.

#### Determination of factors and item reduction

The scree plot and eigenvalue values for each factor guided the evaluation to determine the most parsimonious factor structure. Criteria reported by Costello and Osborne were used for interpretation of the scree plot^16^. Kaiser criterion, which recommends an eigenvalue ≥ 1, was used for factor retention and the assessment of eigenvalues^17^. The interpretation of the scree plot and eigenvalue of each factor determined the number of factors captured by the symptom onset of PASC.

#### Item retention and removal criteria

A priori criteria for item retention were had primary factor loadings ≥ 0.40, secondary factor loadings < 0.30, and did not have primary factor loadings on more than one factor^18^. Items not meeting these criteria were removed individually. The EFA was repeated until all retained items met these criteria.

#### Labeling of factors and internal reliability consistency

The parsimonious factor structure was derived from the EFA and then labeled based content of the items retained^17^. Each factor was assessed for internal reliability consistency to evaluate its contribution to the factor or total score of the symptom onset for PASC.

### Confirmatory factor analysis

#### Determination of model fit

The symptom onset for PASC model identified by the EFA was evaluated for validity using a first-order CFA. We used the following goodness-of-fit indices to determine model fit: *x*^2^, Tucker- Lewis Index (TLI: > 0.90 acceptable, > 0.95 excellent), the Comparative Fit Index (CFI: > 0.90 acceptable, > 0.95 excellent), and the Root Mean Square Error of Approximation (RMSEA: < 0.08 acceptable, < 0.05 excellent)^19^. Paths between error terms were added to enhance the goodness-to-fit of these data to the model based on interpretation of the above modification indices^20^.

## Results

### Sample characteristics

Sample demographics are provided in Table 1. No differences were observed between the EFA, and CFA independent samples based on sex, race/ethnicity, and age. The average time from SARS-CoV-2 infection to completing the questionnaire was 105.2 days (SD 58), and the range was 1–295 days.

**Table 1:** Demographics.

	PASC group			
	EFA group		CFA group	
	N	%	N	%
**Gender**				
Female	2186	85.83	2213	85.48
Male	348	13.66	362	13.98
Non-binary/non-conforming	7	0.27	8	0.31
Unknown	6	0.24	4	0.15
Transgender	0	0.00	2	0.08
**Race**				
White	2086	81.90	2095	80.92
Hispanic or Latinx	231	9.07	231	8.92
Multiracial	64	2.51	93	3.59
Asian/Pacific Islander	53	2.08	60	2.32
American Indian	23	0.90	12	0.46
Black	68	2.67	79	3.05
Middle Eastern	9	0.35	10	0.39
Other	13	0.51	9	0.35
**Age**				
< 18	2	0.08	3	0.12
18–29	198	7.77	213	8.23
30–39	490	19.24	495	19.12
40–49	739	29.01	771	29.78
50–59	684	26.86	699	27.00
60–69	371	14.57	346	13.36
70+	63	2.47	62	2.39
Total	2547	100	2589	100

### Exploratory factor analysis to identify underlying factor structure

**Figure 1:**
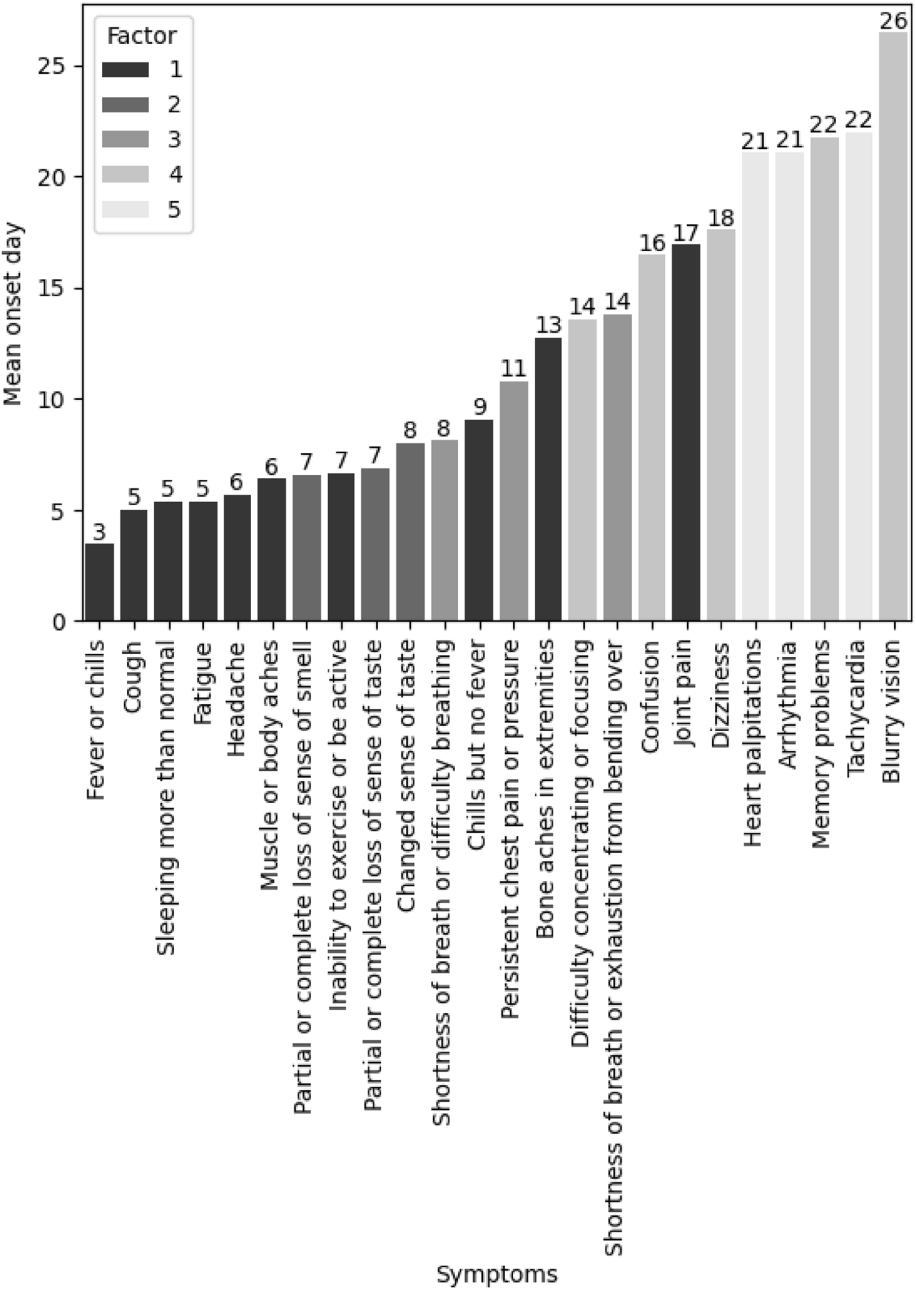
Mean onset of symptoms with factor loading. Bar graph with showing mean symptom onset (in days) and associated factor loading for each symptom. Figure is plotted using R^32^.

**Figure 2:**
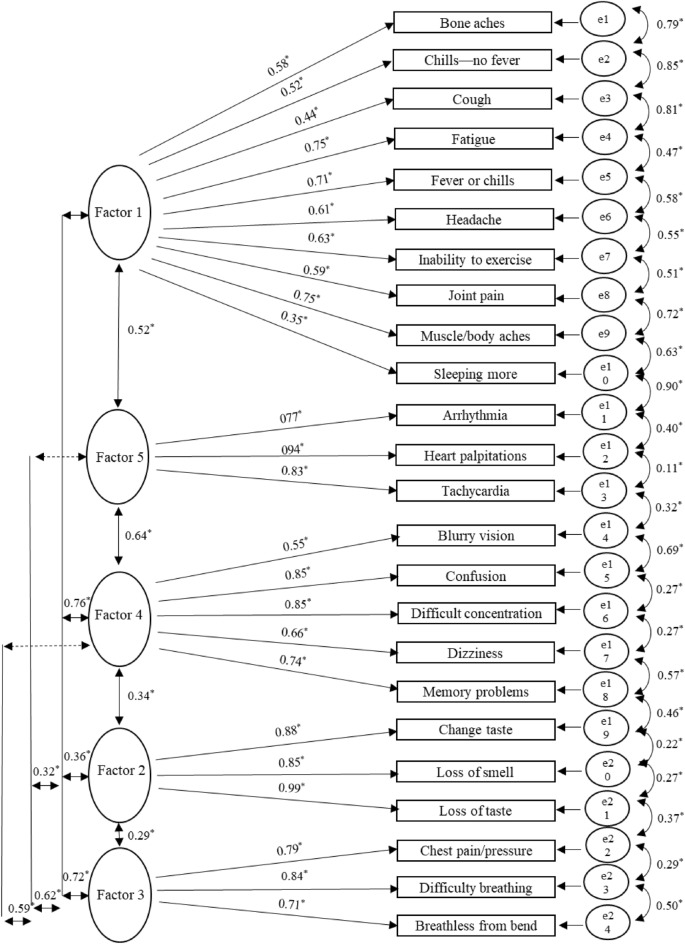
SEM diagram PASC confirmatory factor analysis. Initial SEM model demonstrating 5 factors with symptom loadings denoted. s1, arrhythmia; s2, blurry vision; s3, bone aches in extremities; s4, changed sense of taste; s5, chills but no fever; s6, confusion; s7, cough; s8, difficulty concentrating or focusing; s9, dizziness; s10, fatigue; s11, fever or chills; s12, headache; s13, heart palpitations; s14, inability to exercise or be active; s15, joint pain; s16, memory problems; s17, muscle or body aches; s18, partial or complete loss of sense of smell; s19, partial or complete loss of sense of taste; s20, persistent chest pain or pressure; s21, shortness of breath or difficulty breathing; s22, shortness of breath or exhaustion from bending over; s23, sleeping more than usual; s24, tachycardia. Figure is plotted using R^32^.

The first independent sample of 2547 PASC survivors was used for EFA. In the EFA, the most parsimonious structure—the fewest number of predictors to achieve the greatest explanation—included five factors with a variable number of items per factor (range 3–10). See Table 2 for factor loadings. The mean onset of each symptom and its corresponding factor are presented in Fig. 1. Based on the content of the items retained on each factor and the magnitude of these items’ factor loading, each factor was labeled as follows: Cold and flu-like symptoms (Factor 1), Change in Smell and/or taste (Factor 2), Dyspnea and chest pain (Factor 3), Cognitive-visual symptoms (Factor 4), and Cardiac symptoms (Factor 5). For each factor the item-to-total correlations (range 0.50–0.95) and inter-item correlations (range (0.23–0.92)) are provided in Table 3. See Fig. 2 for the EFA model.

**Table 2:** Individual symptom items retained after the exploratory factor analysis and factor loadings.

PASC symptom items	Factor loading				
	1	2	3	4	5
**Factor 1—cold and flu-like symptoms**					
Muscle or body aches	**0.7**	0.02	0	− 0.06	0.04
Bone aches in extremities	**0.57**	0.08	0.07	− 0.01	− 0.04
Sleeping more than normal	**0.54**	− 0.03	0.12	0.01	0.17
Cough	**0.53**	− 0.01	− 0.12	0.09	0.22
Fever or chills	**0.51**	0.03	− 0.06	0.2	0.13
Joint pain	**0.51**	0.15	0.15	− 0.06	− 0.07
Inability to exercise or be active	**0.48**	− 0.06	0.13	− 0.02	0.24
Fatigue	**0.46**	− 0.06	0.22	0.01	0.23
Headache	**0.46**	0.01	0.21	0.06	− 0.01
Chills but no fever	**0.44**	0.07	− 0.03	0.18	− 0.06
**Factor 2—change in smell and/or taste**					
Partial or complete loss of sense of smell	− 0.06	**0.97**	0.02	0.03	− 0.01
Partial or complete loss of sense of taste	− 0.01	**0.94**	0.01	0.02	0.03
Changed sense of taste	0.2	**0.71**	0.05	− 0.07	0.04
**Factor 3—dyspnea and chest pain**					
Shortness of breath or difficulty breathing	0.03	0.02	**0.86**	0.04	0.01
Persistent chest pain or pressure	0.01	0.12	**0.72**	0.09	− 0.01
Shortness of breath or exhaustion from bending over	0.03	0.21	**0.57**	− 0.08	0.2
**Factor 4—cognitive-visual symptoms**					
Memory problems	− 0.07	0	0.04	**0.93**	− 0.02
Confusion	0.05	− 0.05	0.12	**0.64**	0.15
Difficulty concentrating or focusing	0.08	0.01	0	**0.63**	0.19
Blurry vision	0.11	0.17	− 0.01	**0.54**	− 0.11
Dizziness	0.28	0.03	0	**0.51**	− 0.06
**Factor 5—cardiac symptoms**					
Heart palpitations	− 0.03	0.04	0.06	− 0.02	**0.89**
Tachycardia	0.08	0.06	− 0.02	− 0.03	**0.89**
Arrhythmia	− 0.07	0.03	− 0.02	0.13	**0.8**

N = 2547. The extraction method was principal axis factoring.

Significant values are in [bold].

**Table 3:** Factor-to-factor and interitem correlations.

Item	s3	s5	s7	s10	s11	s12	s14	s15	s17	s23	Total
**Factor 1—cold** and **flu-like symptoms**											
s3	–										
s5	0.28**	–									
s7	0.36**	0.27**	–								
s10	0.29**	0.30**	0.33**	–							
s11	0.41**	0.53**	0.55**	0.36**	–						
s12	0.30**	0.28**	0.30**	0.46**	0.37**	–					
s14	0.28**	0.23**	0.45**	0.60**	0.42**	0.41**	–				
s15	0.67**	0.27**	0.32**	0.28**	0.28**	0.29**	0.35**	–			
s17	0.53**	0.29**	0.37**	0.48**	0.48**	0.49**	0.35**	0.55**	–		
s23	0.27**	0.39**	0.41**	0.68**	0.40**	0.40**	0.62**	0.32**	0.40**	–	
Total	0.62**	0.50**	0.59**	0.69**	0.66**	0.58**	0.67**	0.61**	0.68**	0.71**	–

Item	s4	s18	s19	Total							
**Factor 2—change in smell** and **taste**											
s4	–										
s18	0.75**	–									
s19	0.75**	0.92**	–								
Total	0.78**	0.94**	0.95**	–							

Item	s20	s21	s22	Total							
**Factor 3—dyspnea** and **chest pain**											
s20	–										
s21	0.75**	–									
s22	0.57**	0.66**	–								
Total	0.79**	0.86**	0.71**	–							

Item	s2	s6	s8	s9	s16	Total					
**Factor 4—cognitive-visual symptoms**											
s2	–										
s6	0.35**	–									
s8	0.39**	0.65**	–								
s9	0.44**	0.47**	0.49**	–							
s16	0.57**	0.70**	0.64**	0.54**	–						
Total	0.59**	0.76**	0.74**	0.64**	0.86**	–					

Item	s1	s13	s24	Total							
**Factor 5—cardiac symptoms**											
s1	–										
s13	0.76**	–									
s24	0.76**	0.84**	–								
Total	0.82**	0.90**	0.90**	–							

s1, arrhythmia; s2, blurry vision; s3, bone aches in extremities; s4, changed sense of taste; s5, chills but no fever; s6, confusion; s7, cough; s8, difficulty concentrating or focusing; s9, dizziness; s10, fatigue; s11, fever or chills; s12, headache; s13, heart palpitations; s14, inability to exercise or be active; s15, joint pain; s16, memory problems; s17, muscle or body aches; s18, partial or complete loss of sense of smell; s19, partial or complete loss of sense of taste; s20, persistent chest pain or pressure; s21, shortness of breath or difficulty breathing; s22, shortness of breath or exhaustion from bending over; s23, sleeping more than usual; s24, tachycardia, ** = *p* < 0.001.

### Confirmatory factor analysis confirms pattern of symptom onset

**Figure 3:**
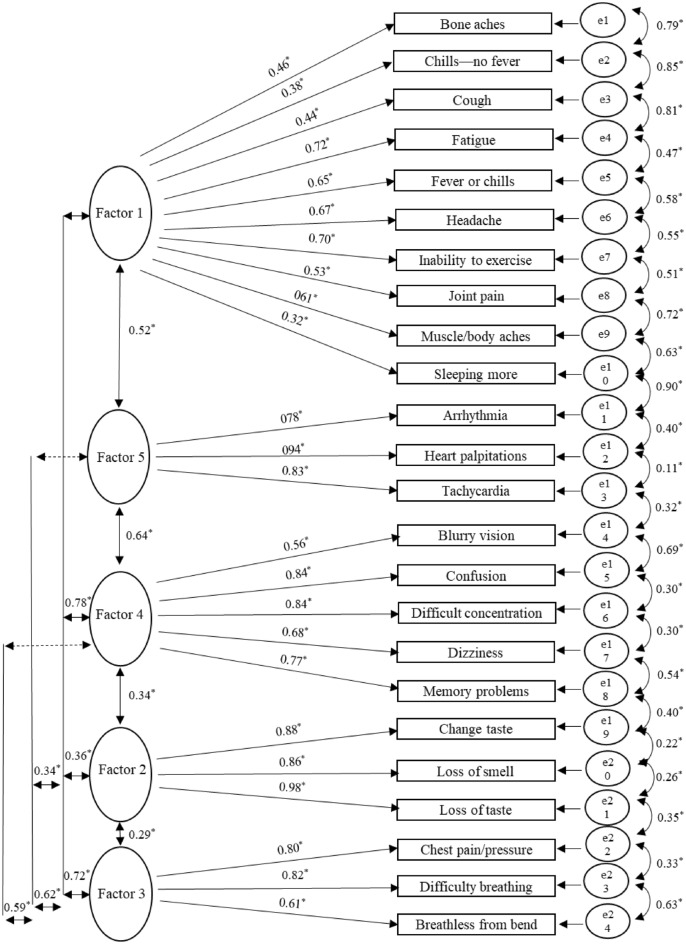
SEM diagram of non-PASC confirmatory factor analysis. Final SEM model demonstrating 5 factors with symptom loadings denoted. s1, arrhythmia; s2, blurry vision; s3, bone aches in extremities; s4, changed sense of taste; s5, chills but no fever; s6, confusion; s7, cough; s8, difficulty concentrating or focusing; s9, dizziness; s10, fatigue; s11, fever or chills; s12, headache; s13, heart palpitations; s14, inability to exercise or be active; s15, joint pain; s16, memory problems; s17, muscle or body aches; s18, partial or complete loss of sense of smell; s19, partial or complete loss of sense of taste; s20, persistent chest pain or pressure; s21, shortness of breath or difficulty breathing; s22, shortness of breath or exhaustion from bending over; s23, sleeping more than usual; s24, tachycardia. Figure is plotted using R^32^.

A five-factor structure was examined for validity with the second independent sample of 2589 PASC survivors. Table 4 provides the goodness-of-fit for the 5-factor structure from the EFA. The initial model is the 5-factor model with all items retained from the EFA. A stepwise approach was then taken whereby paths from the stated items error terms were added sequentially. The final structure model was the best fitting model (*x*^2^ = 513.721, df = 207, *p* < 0.01, TLI = 0.952, CFI = 0.964, RMSEA = 0.024) and the final model is shown in Fig. 3.

**Table 4:** Confirmatory factor analysis model step.

Model step	X^2^	df	*p*	TLI	CFI	RMSEA
**CFA (n = 2589)**						
Initial model	1306.304	242	< 0.01	0.858	0.875	0.042
Correlated s17 and s18	1237.534	241	< 0.01	0.866	0.883	0.04
Correlated s11 and s16	1196.863	240	< 0.01	0.871	0.888	0.04
Correlated s3 and s17	1148.562	239	< 0.01	0.877	0.894	0.039
Correlated s12 and s14	1122.326	238	< 0.01	0.88	0.897	0.038
Correlated s11 and s14	1087.798	237	< 0.01	0.884	0.9	0.038
Correlated s7 and s24	1067.157	236	< 0.01	0.886	0.903	0.037
Correlated s3 and s2	1043.252	235	< 0.01	0.889	0.905	0.037
Correlated s11 and s15	1022.788	234	< 0.01	0.891	0.908	0.037
Correlated s3 and s15	974.742	233	< 0.01	0.897	0.913	0.036
Correlated s5 and s17	950.71	232	< 0.01	0.9	0.916	0.035
Correlated s3 and s5	925.701	231	< 0.01	0.903	0.919	0.035
Correlated s5 and s11	897.836	230	< 0.01	0.906	0.922	0.034
Correlated s10 and s16	869.433	229	< 0.01	0.91	0.925	0.033
Correlated s12 and s23	845.065	228	< 0.01	0.913	0.928	0.033
Correlated s21 and s22	824.629	227	< 0.01	0.915	0.93	0.032
Correlated s9 and s16	806.169	226	< 0.01	0.917	0.932	0.032
Correlated s7 and s8	787.313	225	< 0.01	0.919	0.934	0.031
Correlated s17 and s19	773.107	224	< 0.01	0.921	0.936	0.031
Correlated s10 and s2	755.878	223	< 0.01	0.923	0.938	0.031
Correlated s14 and s18	738.436	222	< 0.01	0.925	0.94	0.03
Correlated s14 and s6	717.827	221	< 0.01	0.927	0.942	0.03
Correlated s15 and s17	697.204	220	< 0.01	0.93	0.944	0.029
Correlated s10 and s17	674.825	219	< 0.01	0.933	0.947	0.029
Correlated s10 and s11	659.193	218	< 0.01	0.935	0.948	0.028
Correlated s7 and s11	642.014	217	< 0.01	0.937	0.95	0.028
Correlated s11 and s24	631.548	216	< 0.01	0.938	0.951	0.028
Correlated s12 and s16	616.712	215	< 0.01	0.94	0.953	0.027
Correlated s12 and s8	605.06	214	< 0.01	0.941	0.954	0.027
Correlated s14 and s17	589.606	213	< 0.01	0.943	0.956	0.026
Correlated s7 and s16	577.972	212	< 0.01	0.944	0.957	0.026
Correlated s7 and s14	565.205	211	< 0.01	0.946	0.959	0.026
Correlated s12 and s21	550.799	210	< 0.01	0.948	0.96	0.025
Correlated s24 and s22	535.47	209	< 0.01	0.95	0.962	0.025
Correlated s14 and s22	523.798	208	< 0.01	0.951	0.963	0.025
Correlated s18 and s21	513.721	207	< 0.01	0.952	0.964	0.024

Tucker-Lewis Index=TLI; CFI = Comparative Fit Index; RMSEA = Root Mean Square Error of Approximation; s1, arrhythmia; s2, blurry vision; s3, bone aches in extremities; s4, changed sense of taste; s5, chills but no fever; s6, confusion; s7, cough; s8, difficulty concentrating or focusing; s9, dizziness; s10, fatigue; s11, fever or chills; s12, headache; s13, heart palpitations; s14, inability to exercise or be active; s15, joint pain; s16, memory problems; s17, muscle or body aches; s18, partial or complete loss of sense of smell; s19, partial or complete loss of sense of taste; s20, persistent chest pain or pressure; s21, shortness of breath or difficulty breathing; s22, shortness of breath or exhaustion from bending over; s23, sleeping more than usual; s24, tachycardia.

## Discussion

To our knowledge this study is one of the first nationwide studies conducted among PASC survivors to document a distinct and significant pattern of symptoms (symptom type and onset) among non-hospitalized persons with confirmed SARS-CoV-2 infection. Most research on PASC has included hospitalized persons with COVID-19. However, there is a far greater number of people who have had SARS-CoV-2 infection, were not hospitalized, and continue to experience symptoms. Findings from this study warrant additional discussion and context to better understand their implications.

We and others have described and characterized a broad range of symptoms associated with SARS-CoV-2 infection, as well as symptoms that persist for months and are a hallmark feature of PASC^4,5,10,14,21^. A limitation shared among many published studies is the use of checklists derived from clinician experience in managing COVID-19. Our initial understanding of symptoms associated with COVID-19 arose from our understanding that SARS-CoV-2 predominantly affected the respiratory system which limited the scope of initial symptom data to the respiratory tract. However, the depth and breadth of symptoms experienced among PASC survivors is just emerging. Indeed, an international study showed PASC survivors experience over 55 symptoms^14^. Moreover, in our own work^4,10^, findings at illness presentation associated with the development of PASC is more extensive than the initial hallmark symptoms of SARS-CoV-2 infection, especially as asymptomatic persons are also at risk.

This study provides new information that points to a distinct and statistically significant pattern of PASC symptoms based on symptom type and symptom onset originally derived from unstructured patient reports. Additionally, this study adds to the science by providing a more granular assessment of PASC symptoms, early post-infection, than is has been measured other longitudinal work that commonly report within set timeframes (e.g., 4-week intervals). Our findings suggests that the evolution of PASC symptoms may follow a predictable pattern inclusive of a long duration (M days post-infection = 105 days, range: 1–295 days). This observation is troubling as it suggests that for some the sequalae of SARS-CoV-2 infection are ongoing and may be life-long. Further, this protracted length of illness suggests a major public health crisis is emerging and is affecting previously healthy and those with pre-morbid conditions alike. Finally, developing and implementing preventative interventions hinges upon understanding the course of the disease in the absence of any intervention. Given that PASC has no treatments or cure, this study documents the temporal order of symptoms during the evolution of the disease, in the absence of intervention, and provides the foundation for preventative and self-management strategies for PASC symptoms.

The confirmed factors (cold and flu like symptoms, change in smell and taste, dyspnea and chest pain, cognitive and visual symptoms, and cardiac symptoms) follow an arguably sequential pattern that could accompany progressive or persistent inflammation accompanied by endothelial activation. Other studies report elevated concentrations of a variety of cytokines (i.e., IL-6, and others) as well as persistent activation of immune cells for many weeks after SARS-CoV-2 infection^22–26^. However, few studies have used biomarkers to correlate biological processes with symptom trajectories. This is an area ripe for exploration among researchers.

This study included mostly women, aged 30–59, who identified as White. While this characteristic the sample may limit generalizability, it is nonetheless important because prior research has also found that PASC appears to be afflicting women to a greater extent than men^4,26^. While the homogeneity of the sample was not planned, such composition is advantageous solely for conducting EFA and CFA; in that, by limiting sample collection from a wide range of populations is recommended to minimize the chance that factors present in one population will be obscured when pooled together with other populations. Further, this research serves as a basis for future comparisons between sex, races, ethnicities, and age groups to guide further research. This study asked PASC survivors to recall symptom type and their onset which is subject to recall bias unless symptoms were recorded via diary or tracking in real time by PASC survivors. However, viewing this weakness should be balanced with weaknesses of other methods of data collection such as clinician recorded symptoms (e.g. in the electronic health record) which has been shown to be discordant with patient reports^27,28^. Ideally future work should be prospective and leverage multiple data sources in real time including patient report and triangulating data sources (e.g., wearable devices, patient report, clinical diagnostics, etc.…) that have been used in other studies. Additionally, prospective cohort data studies are particularly difficult to establish an emerging pandemic as pertinent constructs may not be known.

Second, the survey was conducted among individuals with symptoms following SARS-CoV-2 infection, thus excluding those who were asymptomatic. Moreover, most participants did not require hospitalization and/or supplemental oxygen delivery and would be considered to have mild-COVID-19. Most research in this area has been among hospitalized patients with COVID-19, limiting generalizability to those with less severe or asymptomatic initial presentation^8,11,29^. However, these data should not suggest that persons with asymptomatic infection do not develop PASC. Multiple studies suggest that those with asymptomatic SARS-CoV-2 infection are also at risk for long-term sequelae from COVID-19, although the incidence is unclear. In our work and others, data suggest that persons with PASC likely experience an evolution of symptoms that is unique from persons who do not develop PASC despite SARS-CoV-2 infection^4,10,29^. Model validation utilizing a large sample of patients with SARS-CoV-2 who report prompt resolution of symptoms should be used to validate and understand if, and, or when a bifurcation in symptoms happens.

Third, this survey was conducted earlier in the COVID-19 pandemic when the alpha variant was dominant. Because the delta variant is far more contagious and symptom presentation differs slightly^30^, it is unclear if the pattern reported here would be consistent between the two variants, or among those with the most recent omicron variant. This is an ongoing and active area of research for our team. Also, break through infections are occurring among vaccinated persons, and data suggest that vaccinated individuals are less likely to develop PASC following SARS-CoV-2 infection. However, additional studies are needed to assess PASC among the vaccinated, and should a new variant arise that minimizes the efficacy of current vaccines, the effect of breakthrough infections in the development of PASC would need to be re-evaluated.

Finally, there are potential implications for research and clinical practice^31^. Nursing has an established history in symptom science and our collective expertise is needed at this time to address an unprecedented global health crisis. In addition to advancing the biological and molecular underpinnings of symptoms, Nursing is poised to rapidly re-tool and repurpose symptom management interventions to address symptoms among individuals with PASC. As the science develops around PASC, it may be possible to implement pharmacological and non-pharmacological interventions early during SARS-CoV-2 infection to mitigate potential long- term sequelae. An effective response to this global health crisis will require clinical and research collaboration among nurses to address the symptom burden afflicting the millions and rising numbers of individuals with PASC.

## Conclusion

PASC is a global health crisis that has emerged in response to the novel coronavirus, SARS-CoV-2. This study describes a distinct and statistically significant symptom pattern associated with PASC. We hope that this study will serve as a foundation upon which future studies can further characterize and understand PASC. As the pandemic continues, similar studies are needed to validate our findings and advance the understanding of PASC.

## Data Availability

The data that support the findings of this study are available from Survivor Corps and Dr. Natalie Lambert but restrictions apply to the availability of these data, which were used under license for the current study, and so are not publicly available. Data are however available from the authors upon reasonable request and with permission of Survivor Corps and Dr. Natalie Lambert.
